# Quantitative metabolic analysis of plasma extracellular vesicles for the diagnosis of severe acute pancreatitis

**DOI:** 10.1186/s12951-022-01239-6

**Published:** 2022-01-28

**Authors:** Doudou Lou, Keqing Shi, Hui-Ping Li, Qingfu Zhu, Liang Hu, Jiaxin Luo, Rui Yang, Fei Liu

**Affiliations:** 1 Eye Hospital, School of Ophthalmology & Optometry, School of Biomedical Engineering, Wenzhou Medical University, Wenzhou, 325035 Zhejiang China; 2 The First Affiliated Hospital of Wenzhou Medical University, Wenzhou, 325000 Zhejiang China; 3 Wenzhou Institute, University of Chinese Academy of Science, Wenzhou, 325001 Zhejiang China; 4 Jiangsu Institute for Food and Drug Control, Nanjing, 210019 Jiangsu China

**Keywords:** Severe acute pancreatitis, Early diagnosis, Extracellular vesicles, Metabolomics, Biomarker discovery

## Abstract

**Background:**

Severe acute pancreatitis (SAP) is the most common gastrointestinal disease and is associated with unpredictable seizures and high mortality rates. Despite improvements in the treatment of acute pancreatitis, the timely and accurate diagnosis of SAP remains highly challenging. Previous research has shown that extracellular vesicles (EVs) in the plasma have significant potential for the diagnosis of SAP since the pancreas can release EVs that carry pathological information into the peripheral blood in the very early stages of the disease. However, we know very little about the metabolites of EVs that might play a role in the diagnosis of SAP.

**Methods:**

Here, we performed quantitative metabolomic analyses to investigate the metabolite profiles of EVs isolated from SAP plasma. We also determined the metabolic differences of EVs when compared between healthy controls, patients with SAP, and those with mild acute pancreatitis (MAP).

**Results:**

A total of 313 metabolites were detected, mainly including organic acids, amino acids, fatty acids, and bile acids. The results showed that the metabolic composition of EVs derived from SAP and MAP was significantly different from those derived from healthy controls and identified specific differences between EVs derived from patients with SAP and MAP. On this basis, we identified four biomarkers from plasma EVs for SAP detection, including eicosatrienoic acid (C20:3), thiamine triphosphate, 2-Acetylfuran, and cis-Citral. The area under the curve (AUC) was greater than 0.95 for both discovery (n = 30) and validation (n = 70) sets.

**Conclusions:**

Our data indicate that metabolic profiling analysis of plasma EVs and the screening of potential biomarkers are of significant potential for improving the early diagnosis and severity differentiation of acute pancreatitis.

**Graphical abstract:**

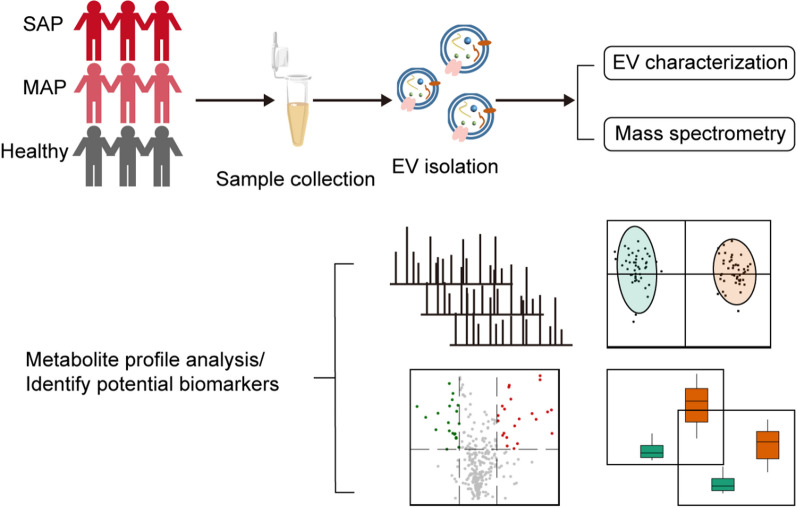

**Supplementary Information:**

The online version contains supplementary material available at 10.1186/s12951-022-01239-6.

## Introduction

Acute pancreatitis is a common global disease and can be defined as mild, moderately severe, and severe [1]. Unlike mild acute pancreatitis (MAP), severe acute pancreatitis (SAP) can often cause pancreatic necrosis, systematic inflammatory disease, and multiple organ dysfunction and failure, with a mortality rate of 20–40%, which is much higher than that of MAP and MSAP (moderate severe acute pancreatitis) [2–4]. MAP may not exhibit typical clinical symptoms, while severe and outbreak cases may kill the patient before diagnosis [5–7]. Therefore, it is vital that we can identify and diagnose SAP rapidly and accurately. However, the clinical diagnostic techniques that are currently available are associated with various limitations. Contrast-enhanced computed tomography (CECT) can play an efficient role in the diagnosis of SAP [8]. CECT can help to characterize pancreatic necrosis, inflammation, and fluid collections, and is, therefore, a highly useful tool for monitoring SAP and facilitating clinical management [9]. However, it requires the injection of contrast agents that are likely to cause further renal burden and affect the microcirculation. Furthermore, CECT may lead to missed or delayed diagnosis since it cannot predict the necrotic process and the ideal time point to perform CECT is up 72 h after the onset of pancreatitis symptoms [10]. Therefore, there is an urgent need to develop new detection methods with higher levels of sensitivity for SAP that is less deleterious to the patient.

Extracellular vesicles (EVs) have been recognized as a potential source of disease markers [11]. By studying EVs, it might be possible to contribute to our understanding of pathogenesis at the molecular level [12, 13]. A range of studies have proved that EVs contained in body fluids are of significant value for the diagnosis of disease [14]. EVs can be released from the inflammatory pancreas and involved organs and directly enter the peripheral blood circulation at a very early stage of the disease, carrying information related to disease progression at a time point well before cellular necrosis. Moreover, EVs are tightly linked to the occurrence and development of acute pancreatitis and organ necrosis, thus rendering these structures an ideal object for the study of pathogenesis and the development of new diagnostic methods [15, 16]. Researchers have demonstrated that EVs released by the pancreas during acute pancreatitis could be found in the plasma of taurocholate-induced rats and carrying specific up-or down-regulated cargo that can exert pro-inflammatory activity [17]. By detecting and analyzing EVs in the peripheral blood, pancreatic necrosis could be detected much earlier than imaging methods. However, most pancreatic disease-related studies of EVs have focused on pancreatic cancer, while acute pancreatitis has received far less research attention [18].

Despite EVs’ various cargos (proteins, nucleic acids, metabolites, et al.), previous studies of EVs have focused predominantly on proteins and nucleic acids; studies relating to small molecule metabolites are rare [17–19]. Compared with proteins and nucleic acids, metabolites are molecules that feature downstream in life processes and can reflect dynamic changes in the body more directly. Therefore, by analyzing the composition and function of exosomal metabolites in the blood, it might be possible to diagnose pancreatitis much earlier and provide rapid interventional treatment. Metabolomics has significant potential for measuring differential metabolites that are created by pathophysiological changes and have become a powerful tool for the discovery of diagnostic biomarkers for pancreatic disease [20, 21]. Over the past few years, metabolomics has been used to identify the etiology of acute pancreatitis [22] and judge the severity of this disease [23]. In addition, metabolomics has become a potential tool to differentiate acute pancreatitis from chronic pancreatitis, and pancreatic cancer, as well as other diseases of the digestive system [24–28]. For example, Huang et al. distinguished three types of acute pancreatitis (biliary acute pancreatitis, hyperlipidemia acute pancreatitis, alcoholic acute pancreatitis) by gas chromatography-mass spectrometry-based metabolomics [29]. Xiao et al. also successfully identified potential biomarkers for acute pancreatitis and some relevant pathways by analyzing serum metabolomic profiles [23]. However, only a few metabolomics studies have focused on pancreatitis. Furthermore, these previous studies were more focused on differences between inflammatory and healthy samples, rather than between severe and mild cases, and were often limited to small clinical sample sizes or based on animal models [22, 24]. Moreover, to the best of our knowledge, no previous study has applied metabolomics analysis to plasma EVs to study acute pancreatitis.

**Fig. 1:**
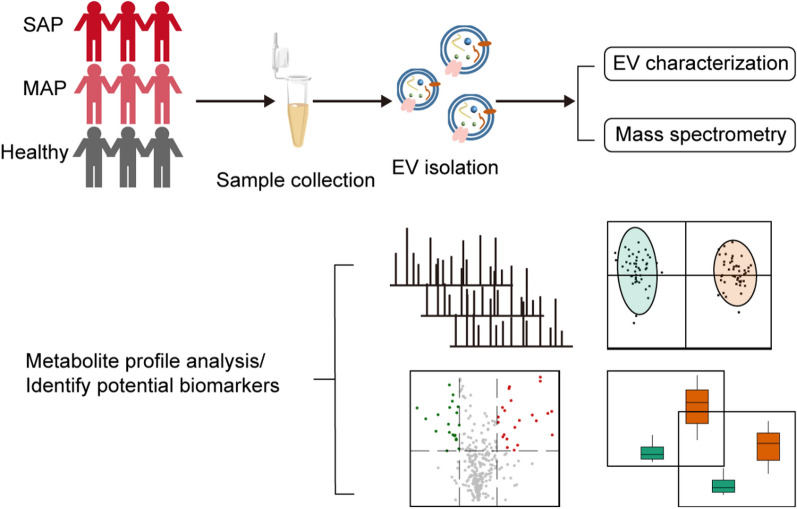
The schematic diagram of the EV isolation, characterization, and metabolomics analysis for SAP detection. EVs were isolated from three groups of plasma samples (healthy controls, SAP, and MAP) for the downstream characterization and LC-MS analysis. Then the metabolic differences of EVs were compared and the potential biomarkers were identified

In this study, we used a home-constructed EV ultrafast isolation system named EXODUS to isolate EVs with both high purity and high yield from the plasma of patients with pancreatitis [25]. Several approaches were used to observe and distinguish the typical characteristics of EVs, including morphology and size distribution. Metabolite profile analysis was carried out to evaluate the characteristics of metabolites from plasma EVs and to describe the overall metabolic differences between diseased and healthy samples. Finally, we identified several potential metabolic biomarker candidates that can be used to distinguish between SAP and MAP; our overall aim was to provide a new perspective for the diagnosis of SAP (Fig. 1).

## Materials and methods

### Subject information

All the subjects were recruited from the First Affiliated Hospital of Wenzhou Medical University. This research was approved by the hospital’s ethics committee and each subject’s informed consent was obtained. A total of 150 plasma samples from 150 subjects were collected, including 50 from SAP patients, 50 from MAP patients, and 50 from healthy people. All the patients were diagnosed according to the revised Atlanta classification and definitions [1]. The blood samples of all the patients were collected as soon as they were admitted to hospital.

### Isolation of EVs from plasma

Human plasma was centrifugated at 1500 g (4 ℃) for 15 min within 2 h after collection. The supernatant was collected and centrifuged again under the same condition to further remove the platelets and cell debris. Next, a portion of 20 µL supernatant was diluted to 5 mL by PBS and filtered by a 0.22 μm membrane. Then EVs were isolated from this supernatant by using the EXODUS system [25]. The concentrated solution was washed with 1 mL PBS twice to further remove impurities. Finally, the isolated EVs were resuspended to 200 µL with PBS and stored at − 80℃ for further analysis. All the 1 × PBS used in this research was filtered by 0.22 μm membrane beforehand.

### Characterization of EVs

*Transmission electron microscopy (TEM)* 20 µL of EVs were mixed with 20 µL of 4% paraformaldehyde and dropped onto hydrophobic parafilm, then a piece of the formvar carbon-coated copper grid (Zhongkejingyi, China) was floated onto the mixture for 20 min. Excess liquid was removed with filter paper and the grid was air-dried for 1 h. The grids were negatively stained with 2% uranyl acetate for 30 s and the excess dye was removed. After the grids were dried, the images were obtained with a transmission electron microscope (Helios NanolabDualBeam, FEI, USA) at an acceleration voltage of 80 kV.

*Nanoparticle tracking analysis (NTA)* The particle concentration and size distribution were measured by NTA (NanoSight NS300, Malvern, UK). EVs were diluted with 1× PBS to ensure that the number of particles per frame was between 20 and 100. The camera level was set to be 15 and the temperature was at 25.0 ℃. The diluted samples were infused automatically using a micropump and 6 videos of 30 s each were acquired. The obtained data were analyzed with NTA software (version 3.4) with a detection threshold set at level5.

*Sample preparation and metabolite extraction* After lyophilization, 1 mL of 70% methanol was added toEVs. Three freeze-thaw cycles were operated to accelerate the release of metabolites from vesicles as much as possible. In each cycle, samples were vortexed for 30 s, then put in liquid nitrogen for 5 min, and unfroze on ice for 3 min. After vortex mixing for another 30 s, samples were treated with 30 Hz ultrasound at 4 ℃ for 3 min. Then samples were vortexed for 30 s and centrifuged at 12,000 rpm for 10 min (4 ℃). The supernatant was transferred to a fresh tube and concentrated at 4 ℃. 150 µL of 70% methanol was added to the concentrates. The mixture was vortexed for 30 s and centrifuged at 12,000 rpm for 10 min (4 ℃). The final supernatant was collected for the metabolomics analysis.

*Metabolic profiling analysis based on LC-MS/MS* For sensitive and comprehensive metabolic profiling analysis of EVs, LC-MS/MS was used. Concretely, the data were acquired with an LC-ESI-MS/MS system (UPLC, Shim-pack UFLC SHIMADZU CBM30A, Japan; MS, QTRAP® System, SCIEX, USA). The main LC-MS analytical conditions were as follows. 2 µL of metabolite extracts were injected into the LC-MS system for analysis. The separations were operated with the ACQUITY UPLC HSS T3 C18 (1.8 μm, 2.1 mm × 100 mm, Waters, USA). Mobile phase A contained 0.04% (v/v) acetic acid in aqueous solution and mobile phase B contained 0.04% (v/v) acetic acid in acetonitrile solution. The flow rate was set to be 0.4 mL/min and the column temperature was kept at 40 ℃. The gradient elution program was performed with aqua/acetonitrile (95: 5 v/v at 0 min, 5:95 v/v at 11.0 min, 5:95 v/v at 12.0 min, 95:5 v/v at 12.1 min and 95:5 v/v at 14.0 min).

LIT and triple quadrupole (QQQ) scans were acquired on a triple quadrupole-linear ion trap mass spectrometer (QTRAP), QTRAP® LC-MS/MS System, equipped with an ESI Turbo Ion-Spray interface, operating in positive and negative ion mode, and controlled by Analyst 1.6.3 software (Sciex). The main operating parameters were as follows. The source temperature was 500 ℃. The ion spray voltage (IS) was 5500 V (positive) and − 4500 V (negative). The ion source gas I (GSI), gas II (GSII), and curtain gas (CUR) were set at 55, 60, and 25 psi, respectively. The collision-activated dissociation (CAD) was high. Each ion pair was detected based on optimized declustering potential (DP)and collision energy (CE). Instrument tuning and mass calibration were performed with 10 and 100 µmol/L polypropylene glycol solutions in QQQ and LIT modes, respectively. A specific set of MRM transitions were monitored for each period according to the metabolites eluted within this period.

*Data processing and statistical analysis* The software analyst was used to process the mass spectrum data. The integration and calibration of chromatographic peaks were carried out by using MultiQuant. Orthogonal partial least squares-discriminant analysis (OPLS-DA) was applied to show the overall metabolic differences among different groups. The parameters including fold change (FC), *p*-value, and variable importance in projection (VIP) were used for screening out differential metabolites and further analysis.

## Results and discussions

### Clinical characteristics of the subjects

A total of 150 plasma samples were used to identify metabolic differences between SAP (50 samples), MAP (50 samples), and healthy controls (50 samples). The clinical characteristics were shown in Additional file 1: Table S1. There was no significant difference in the duration of abdominal pain when compared between groups (*p* > 0.05).

### Isolation and characterization of EVs

For each sample, EVs were isolated from 20 µL of plasma by using EXODUS. Each plasma sample was diluted 250-fold to eliminate the adverse effects of clogging and aggregation with regards to isolation time and purity. As shown in Additional file 1: Fig. S1, the isolated EVs were characterized by TEM and NTA. TEM showed that the EVs had a typical cup-shaped structure. The size distribution of the isolated EVs presented a smooth unimodal curve according to NTA, with peaks ranging from 80 to 100 nm. There were no significant differences between the EVs from SAP, MAP, and healthy controls, with regards to mean diameter and size distribution.

### Differential metabolic profiles of EVs isolated from SAP, MAP, and healthy control groups

**Fig. 2:**
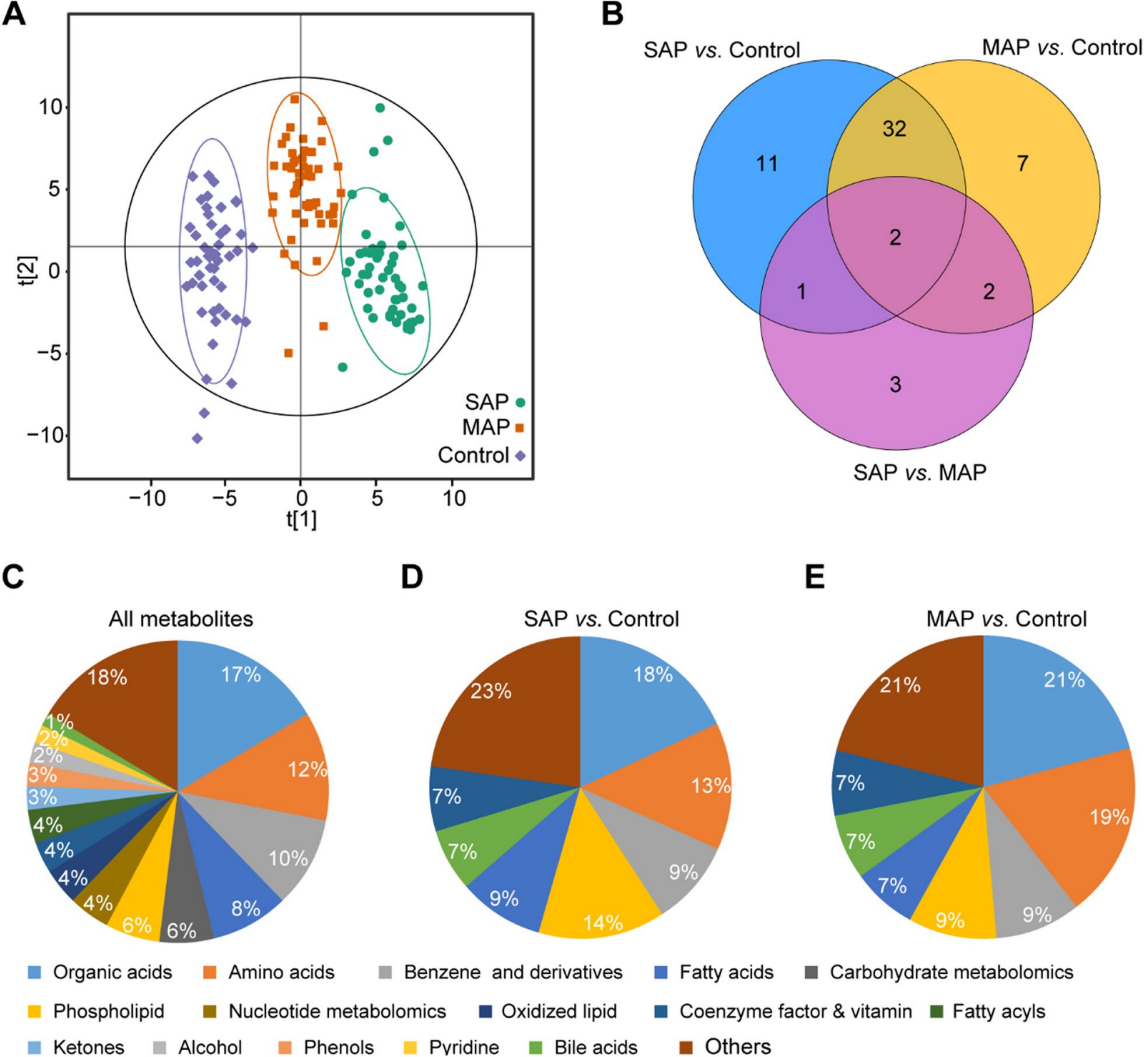
Characterization of metabolic profiles from samples of SAP, MAP, and healthy controls. **A** The OPLS-DA analysis and **B** the Venn diagram of the differential metabolites between the SAP, MAP, and healthy control groups. Composition of (**C**) the overall detected metabolites, and the differential metabolites found in the comparisons of (**D**) SAP *vs*. healthy control and (**E**) MAP *vs*. healthy control, respectively

A total of 313 metabolites were successfully detected by widely targeted metabolomics based on ultra-performance LC-MS/MS. The OPLS-DA model, which is sensitive to less correlated variables, was used to show the differential abundance of metabolites across all three groups. Among the 313 detected metabolites, differential metabolites were screened out according to the FC value (≥ 1.5). There was a large variety of metabolites in the EVs isolated from SAP, MAP, and healthy control groups (Fig. 2 A). Correspondingly, the expression of metabolites in EVs isolated from the SAP and MAP groups were both significantly different from those in the healthy control group (Fig. 2B). There were obvious differences between exosomal metabolites from patients and healthy samples, while the metabolite profiles of SAP and MAP groups showed both overlaps and differences; this requires further analysis. The metabolites detected were mostly organic acids and their derivatives, amino acid metabolomics, benzene and substituted derivatives, carbohydrate metabolomics, and lipid species from fatty acids, phospholipids, and oxidized lipids (Fig. 2C), thus indicating that these metabolites were common components of EVs. Among the differential metabolites, the most abundant metabolite classes were organic acids, amino acids, benzene and its derivatives, phospholipids, fatty acids, bile acids, and coenzyme factors and vitamins; these accounted for most of the detected differential metabolites (Fig. 2D and E). The proportions of fatty acids among the differential metabolites in SAP samples were higher than those in MAP, while more organic acids, amino acids, and phospholipids differed in MAP. Differences in EV metabolites between the SAP and MAP groups were smaller than those between disease samples and healthy control samples.

**Fig. 3:**
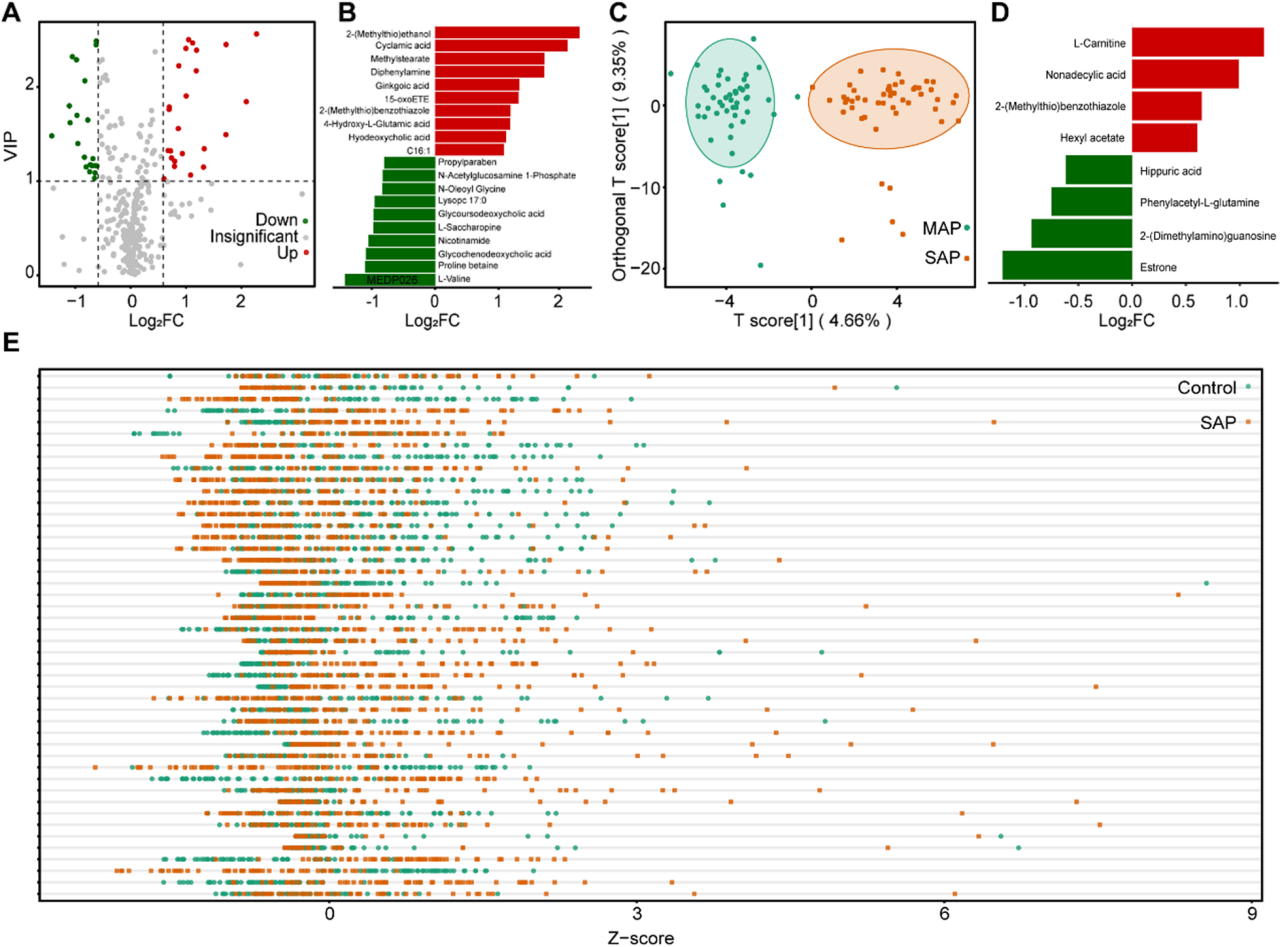
Analysis of the differential metabolites in comparison to SAP, MAP, and healthy controls. **A** The volcano plot showing the differential metabolites in SAP EVs compared to healthy control; **B** The top 20 differential metabolites of the SAP and healthy control groups based on the FC values; **C** The OPLS-DA score plot for distinguishing SAP and MAP groups; **D** The top 8 differential metabolites of the SAP and MAP groups. **E** The z-score plot of differential metabolites in the SAP and MAP groups

It is well established that pancreatic disease can influence the metabolism of amino acids (Fig. 3B and Additional file 1: Fig. S2C). For example, we found that valine was significantly down-regulated in both the SAP and MAP groups. Pancreatic disease is closely associated with the down-regulation of valine. Furthermore, the pathway underlying the biosynthesis of valine is involved in the pathogenesis of acute pancreatitis [24]. In addition, valine is a component of pancreatic juice [30]; pancreatic insufficiency might be responsible for valine reductions. Another example is the up-regulation of glutamic acid’s derivatives, which is also a symptom of pancreatic disease [28]. Bile acids and choline were also found to markedly alter in diseased samples. Glycoursodeoxycholic acid was down-regulated, which might interfere with lipid digestion. The toxic effect of bile acids on acinar cells also induces the formation of pro-inflammatory mediators, although the clinical significance of this process has yet to be determined [7]. We detected an overall reduction in the levels of phosphatidylcholines in pancreatitis samples. Choline metabolism plays a significant role in inflammatory responses and can change various pancreatic diseases [28].

Previous studies have also demonstrated that changes in bile acids, phosphatidylcholine, and amino acids occur in acute pancreatitis [27, 31, 32], thus confirming our present findings. However, few researchers have investigated differences in metabolites between severe and mild cases.

### Differential EV metabolites between SAP and MAP

Compared to EVs from healthy controls, a total number of 46 and 43 differential metabolites were detected in SAP-EVs and MAP-EVs, respectively. Of these, 34 metabolites were shared by the SAP and MAP groups (Figs. 2B and 3A, and Additional file 1: Fig. S2A). The metabolites showing the greatest changes in samples of disease patients included organic acids, bile acids, amino acids, benzene, substituted derivatives, and others (Fig. 3B and Additional file 1: Fig. S2B). It was clear that there were significant changes in metabolites associated with inflammation, the pancreas, and digestion.

We further evaluated the metabolic characteristics of EVs between SAP and MAP groups and investigated the metabolic characteristics of SAP- and MAP-EVs. OPLS-DA score plots revealed overall differences in metabolites between the SAP and MAP groups (Fig. 3C). The metabolic profiles of EVs were compared between the SAP and MAP groups, and we found that these groups shared many differential metabolites when compared to healthy control samples. Also, some metabolites showed unique changes only in the SAP or MAP groups (Fig. 2B). A total of eight differential metabolites were detected and showed significant differences (Fig. 3D), including estrone, non-adecylic acid, phenylacetyl-L-glutamine, L-carnitine, hippuric acid, 2-(dimethylamino)-guanosine, hexyl acetate, and 2-(methylthio)benzothiazole. We also created a Z-score plot to present the overall differences between the two groups (Fig. 3E).

Based on these results, it was clear that the metabolomic characteristics of EVs can reflect the metabolic characteristics of patients with SAP and MAP. Furthermore, the unique differential metabolites found in patients with SAP indicated the distinct roles of these metabolites in the genesis and development of SAP. Consequently, there is a strong possibility that EV-carried metabolites may be used to identify new biomarkers for the diagnosis of SAP.

### Selecting potential biomarkers for distinguishing SAP and MAP

**Fig. 4:**
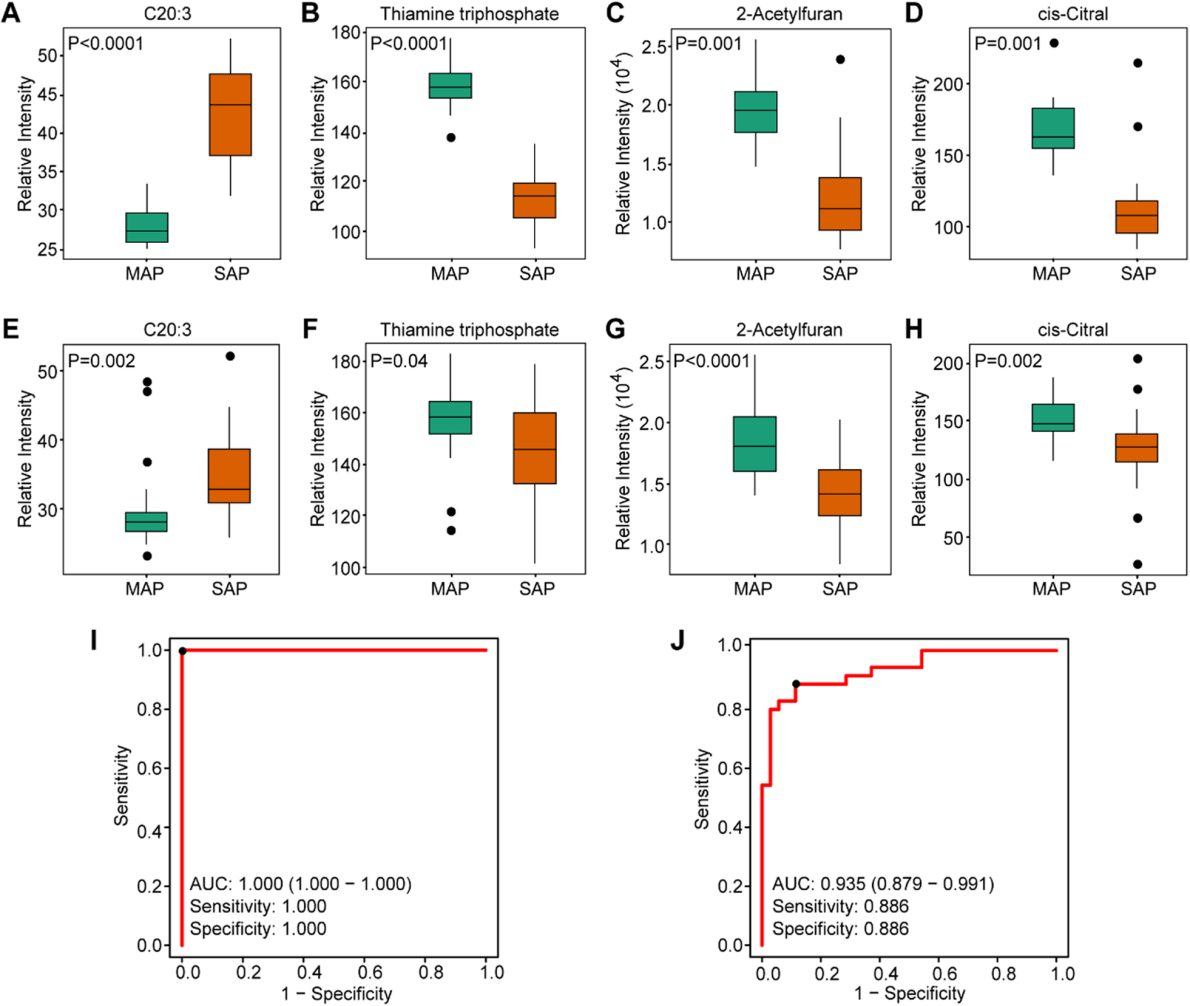
Identification of metabolite biomarkers for distinguishing SAP and MAP. Relative intensities of the defined biomarker candidates in the discovery set (**A–D**) and the validation set (**E–H**). (**I–J**) ROC performance based on the selected metabolic markers (C20:3, thiamine triphosphate, 2-acetyl furan, and cis-citral) in the discovery and validation sets, respectively

Plasma EVs were further selected as potential metabolic biomarker cargo to distinguish between samples with SAP and MAP. According to OPLS-DA analysis, samples from the SAP and MAP groups separated well (Additional file 1: Fig. S2C). We performed random forest analysis, in which 30 cases (15 SAP and 15 MAP samples) were used as a discovery set to identify metabolic biomarker candidates for the diagnosis of SAP, and 70 cases (35 SAP and 35 MAP samples) were assigned as a validation set. In the discovery set, the VIP value was the main parameter used to assess the contribution of different variables to the classification of data, in combination with the FDR value. We identified 9 metabolites that satisfied the screening conditions with VIP value> 1.0 and FDR < 0.05 (Additional file 1: Table S2).Then the 9 differential metabolites were ranked in order of VIP value from highest to lowest, and the first 4 metabolites were identified as potential biomarkers between the SAP and MAP groups: cis-11,14,17-eicosatrienoic acid (C20:3), thiamine triphosphate, 2-acetyl furan, and cis-citral (Fig. 4A–D).These candidates also showed remarkable differences when compared between SAP and MAP samples in the validation set (Fig. 4E–H). Receiver-operating characteristic curve (ROC) analysis was then used to further assess the performance of the candidates. The area under the curve (AUC) values for each candidate was> 0.90 in the discovery set and > 0.68 in the validation set (Additional file 1: Fig. S3). More significant differences were obtained by combining the candidates rather than using the metabolites individually (Fig. 4I, J). When metabolites were combined, the AUC values increased to 1.00 and 0.935 for the discovery and validation sets, respectively. Furthermore, the sensitivity and specificity were both 1.00 in the discovery set and 0.886 in the validation set. Hence, the changes in expression levels of the four candidates showed significantly difference between SAP and MAP samples.

## Conclusions

We characterized the metabolite signatures of EVs isolated from patients’ plasma samples. We also analyzed metabolite profiles from patients with acute pancreatitis, which indicated the clear potential for EV-related metabolomics to distinguish between diseased and healthy EVs and facilitate the discovery of biomarker candidates. Nevertheless, the study of metabolomics in pancreatitis remains in its primary stages. The uncertainty underlying the precise etiology of acute pancreatitis creates a limitation for treatment in the early stages of the disease. Therefore, additional studies are now needed to explore the molecular mechanisms of acute pancreatitis at the EV level. The various etiologies of acute pancreatitis, which mainly include biliary, alcoholic, and hyperlipidemic acute pancreatitis, could also be reflected in the observed differences in metabolites. Future studies should include a more comprehensive classification and analysis of such samples. In addition to diversity relating to etiology, it can be easy to confuse acute pancreatitis with many other diseases of the digestive system. Therefore, it is necessary to improve the timing, sensitivity, and accuracy of diagnosis. For more effective and precise identification of the severity, further study on the metabolome differences between MSAP and SAP is also needed. In summary, the use of metabolomics to study plasma EVs provides us with a new opportunity to improve the diagnosis, treatment, monitoring, and prognosis of acute pancreatitis and may help us to develop a deeper understanding of the precise mechanisms involved.

## Supplementary Information

**Additional file 1**: The TEM and NTA characterization results of isolated EVs; the volcano plot and top 20 differential metabolites between MAP and healthy control groups; the OPLS-DA analysis of SAP and MAP samples; the ROC analysis of the four biomarker candidates in the discovery and validation sets.

## Data Availability

All data generated or analyzed during this study are included in the article and additional files.
