# Supplementary material for: Quantitative metabolic analysis of plasma extracellular vesicles for the diagnosis of severe acute pancreatitis
Source: J Nanobiotechnology. 2022 Jan 28;20:52. doi: 10.1186/s12951-022-01239-6 (PMC8796348; doi:10.1186/s12951-022-01239-6)
Supplement: Supplementary file 1 — Additional file 1: The TEM and NTA characterization results of isolated EVs; the volcano plot and top 20 differential metabolites between MAP and healthy control groups; the OPLS-DA analysis of SAP and MAP samples; the ROC analysis of the four biomarker candidates in the discovery and validation sets. [file 12951_2022_1239_MOESM1_ESM.pdf]

Quantitative metabolic analysis of plasma extracellular vesicles for the diagnosis of severe acute pancreatitis

Doudou Lou[1,4#], Keqing Shi[2#], Hui-Ping Li[2#], Qingfu Zhu[1], Liang Hu[1], Jiaxin Luo[1], Rui Yang[1], Fei Liu[2,3]*

[1]Eye Hospital, School of Ophthalmology & Optometry, School of Biomedical Engineering, Wenzhou Medical University, Wenzhou, Zhejiang 325035, China
[2]The First Affiliated Hospital of Wenzhou Medical University, Wenzhou, Zhejiang 325000, China
[3]Wenzhou Institute, University of Chinese Academy of Science, Wenzhou, Zhejiang 325001, China
[4]Jiangsu Institute for Food and Drug Control, Nanjing, Jiangsu210019, China

# Dr. Doudou Lou, Dr. Keqing Shi, and Dr. Hui-Ping Li contributed equally to this work.

*feiliu@wmu.edu.cn (Fei Liu)

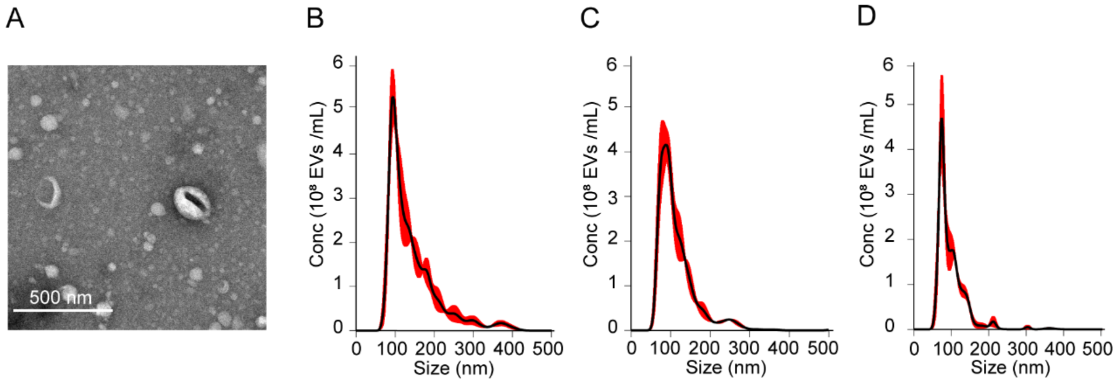

Figure S1. (A) The TEM image of the EVs isolated from human plasma. The NTA results of EVs isolated from human plasma of (B) SAP, (C) MAP, and (D) healthy controls.

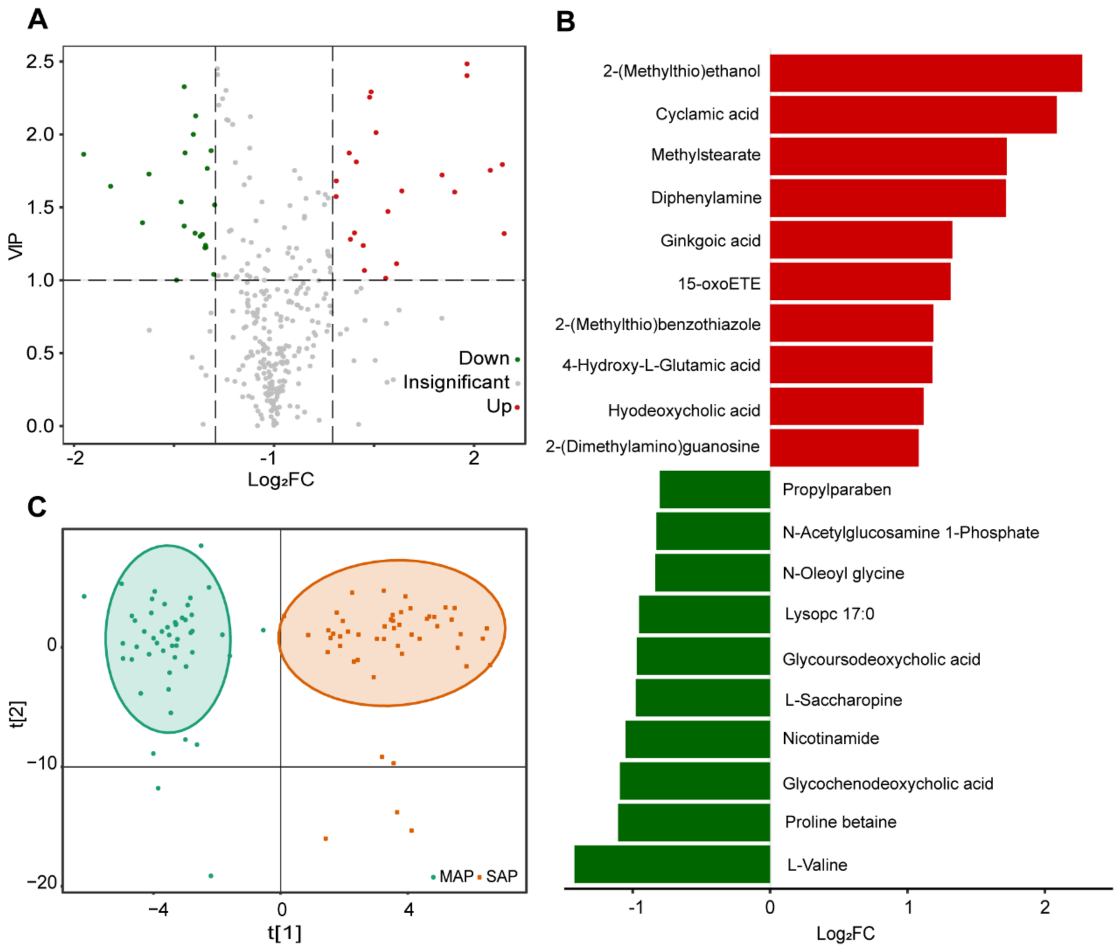

Figure S2. (A) The volcano plot of metabolites up-and down-regulated in MAP exosomes compared to healthy control; (B) The top 20 differential metabolites of the MAP and healthy control groups based on the Fc value; (C) The OPLS-DA analysis of SAP and MAP samples.

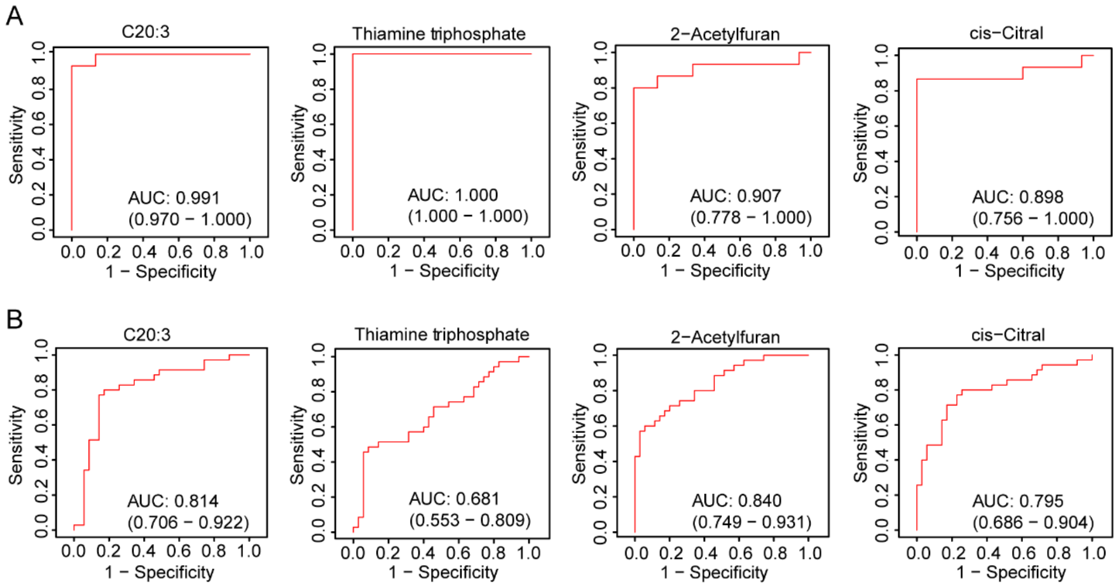

Figure S3. ROC analysis of each biomarker candidate (C20:3, thiamine triphosphate, 2-acetylfuran, and cis-citral) in the discovery set (A) and validation set (B), respectively.

Table S1. Demographic of plasma samples from pancreatitis patients.

	Healthy control (n=50)	Pancreatitis (n=100)	Pancreatitis	
			SAP (n=50)	MAP (n=50)
Men No. (%)	/	64(64%)	35(70%)	29(58%)
Women No. (%)	/	36(36%)	15(30%)	21(42%)
Median age (range)	/	47 (17-85)	45 (21-85)	50 (17-81)

SAP (n=50)	Creatinine*	Oxygenation index (PaO2/FiO2)*
	2	37

Etiology	Biliary	Hyperlipidemic	Others
SAP (n=50)	8	14	28
MAP (n=50)	12	18	20

	Discovery set		Validation set	
	SAP (n=15)	MAP (n=15)	SAP (n=35)	MAP (n=35)
Men No.	6	8	29	21
Women No.	9	7	6	14
Median age (range)	44 (22-85)	51 (23-81)	47 (21-77)	48 (17-76)

* According to the 2012 Atlanta criteria, abnormal serum creatinine and oxygenation index indicates possible renal injury and respiratory injury, respectively.

People who were diagnosed with acute pancreatitis according to the 2012 Atlanta criteria were all included. The judgment of MAP and SAP was also based on this criteria.

Table S2. The potential metabolic biomarker candidates for the distinguishing of SAP and MAP (VIP ＞ 1.0, FDR ＜ 0.05).

Name	Class	VIP	HMDB
Cis-11,14,17-Eicosatrienoic Acid (C20:3)	Lipids_Fatty Acids	3.0332	HMDB60039
Thiamine Triphosphate	Co-Others Enzyme Factor & vitamin	3.0095	HMDB01512
Palmitaldehyde	Lipids Fatty Acids	3.0201	HMDB01551
4-Ethylbenzoic Acid	Benzene and substituted derivatives	2.4016	HMDB02097
2-Acetylfuran	Heterocyclic compound	3.2201	HMDB33127
cis-Citral	Aldehyde	2.5658	HMDB35092
alpha-Terpinene	Terpenoid	2.8518	HMDB36995
2-(Methylthio)benzothiazole	Benzene and substituted derivatives	2.9464	--
Pulegone	Ketones	2.0081	HMDB0035604
